# It still takes a village: an epidemiological study of the role of social supports in understanding unexpected health states in young people

**DOI:** 10.1186/s12889-015-1636-2

**Published:** 2015-03-27

**Authors:** Colleen Davison, Valerie Michaelson, William Pickett

**Affiliations:** Department of Public Health Sciences, Carruthers Hall, 62 Fifth Field Company Lane, Queen’s University, Kingston, ON K7L 3N6 Canada; Department of Public Health Sciences and School of Religion, Theological Hall, Queen’s University, Kingston, Ontario K7L 3N6 Canada

**Keywords:** Adolescent, Child, Epidemiology, Health status, Social supports, Socio-economic status

## Abstract

**Background:**

This study of adolescent Canadians examines two groups who are anomalous in their health experiences: (1) those with perceived low affluence yet who perceive themselves to have excellent general health status; (2) those of perceived high affluence but who are reporting poor health status. Our hope was to explore the role of social supports in explaining such anomalies. We hypothesized that cumulative levels of social support available to these young people would have an influence on their perceived health status, with more support being associated with better self reported health.

**Methods:**

Young people (n = 26,078 from 436 schools) aged 11–15 years were administered a general health survey in classroom settings during the 2009–10 academic school year. Descriptive and regression-based cross-sectional analyses (with an affluence-social support interaction term) were used to relate both individual and cumulative levels of social support in homes, neighborhoods, schools, and peer groups to self-reported health status.

**Results:**

Social supports and their cumulative availability indeed were strongly related to perceived health, with more supports being associated with better self-perceived health. Less affluent children were much more likely to report excellent health in the presence of numerous social supports. More affluent children were much more likely to report poor health in the absence of such supports. The strength and dose-dependent nature of the findings were consistent and striking.

**Conclusions:**

Study findings from this large, contemporary and national analysis affirm the importance of social supports as potential determinants of health for young people from both high and low affluent groups. Conceptually, findings affirm the wisdom of the ancient principle: “it takes a village to raise a child”.

## Background

Of the many factors that contribute to positive health states in young people, wealth is one of the most influential. Increased wealth and associated social advantage are known to protect children from disease, as observed in virtually all population contexts [1]. Conversely, the pervasive effects of poverty on the health of children have been observed consistently regardless of the measure of mortality or morbidity employed [2,3]. Therefore, if a wealthy child fails to thrive in terms of their health and well-being, or if a young person from a situation of financial hardship reports optimal health, it is paradoxical and unexpected [4].

However unexpected, it is clear that such social anomalies exist. Development of a deeper understanding of these anomalies and the mechanisms that underlie their occurrence can provide important clues about health and its determinants in child populations [4]. One explanatory mechanism for a health-wealth pattern deviance may be the social support networks that are available to a young person. Social supports have demonstrated health effects as observed across population subgroups defined by age, sex, ethnic background, and level of affluence [5,6].

One of the earliest definitions of social support was provided by Cobb [7], who understood it as an individual’s belief that s/he is cared for, loved, valued and that s/he belongs to a network of communication and mutual obligation. More recently, it has been defined as “interactions with family members, friends, neighbours, peers and health care providers that may provide instrumental informational, emotional and appraisal support” ([8], p. 6) and its dynamic and multidimensional nature have been recognized [9,10]. In a World Health Organization international review, Barker [11] suggested that social supports represent “a range of interpersonal relationships or connections that have an impact on the individuals’ functioning, and generally include support provided by individuals and by social institutions” (p.3) [11].

Social supports have demonstrated effects on the health of young people. When present, they can protect children from harmful stressors [12]. Indeed, even perceptions that social supports are available alone can buffer negative or stressful events [13]. Barker [11] notes that the belief that young people have access to support is actually more critical than whether or not they use this support. Within adult populations, higher levels of social support have been related to better overall health [14], including better outcomes for those with oncological and cardiovascular diseases [15] and multiple sclerosis [16] as well as better overall physical health outcomes [17]. It also relates to emotional health outcomes, including the alleviation of psychological responses to stress [18] and lower loneliness [19]. Positive social supports also relate to behaviours such as increased exercise, eating a more healthy diet, or deciding not to smoke [20]. Emotionally, such supports may also help a person feel more in control of his or her environment and in turn lead to increased feelings of self-worth and well-being [21]. Notwithstanding these positive effects, negative consequences are also possible [11]. For example, an adolescent may turn to a peer for help and support, who instead encourages risky or anti-social behaviour [11].

Contexts in which social supports arise are important, and start with home environments. Communication practices within family units consistently relate to child health outcomes [22]. However, even when such communication is sub-optimal, other family-based supports such as engagement in family dinners [22] and simply knowing that one is living within a food secure household [23] can make a positive difference. Neighbourhoods where young people grow up also significantly influence adolescent health outcomes, as demonstrated by recent investigations of the influence of social capital [24]. Furthermore, group involvement in neighbourhood settings, and in particular sports involvement, are each related to positive health outcomes [25]. Other research has shown that positive social climates at schools [26] and within student bodies [27] can be equally influential on adolescent health outcomes, and the influence of supportive peers is obvious, strong, and well-established [28,29].

Much of the existing adolescent literature has focused upon the relative influence of individual types of supports in protecting young people from adverse health outcomes. A large body of literature examines the relationship between help seeking behaviours, social supports and specific adolescent health problems, e.g., HIV, mental health, and sexually transmitted infections [11]. What are less well established are the cumulative effects of such supports on health, including supports that exist in different social contexts related to wealth.

Our research group had a unique opportunity to explore the role of social supports in fostering the health of adolescents in a national and contemporary study of young people in Canada. Our first focus was to identify groups of children in different wealth categories whose behaviour was anomalous from what was expected – those of low affluence who thrived in terms of their health, and those with wealth who did not. Next, we explored whether the cumulative presence or absence of social supports in home, neighbourhood, school and peer-group environments could explain the presence of these anomalies. Formal study of these issues provides evidence which may assist in refining social theory, as well as inform practical interventions to build supportive contexts for young people from different population sub-groups.

## Methods

### Study population and procedures

Our study was based upon the 2010 Canadian Health Behaviour in School-aged Children study (HBSC). HBSC involves written health surveys conducted in classroom settings, with a focus on the early adolescent years (ages 11–15). It is administered every four years, most recently in 2009–10, following a common international protocol [30].

The 2009–10 (Cycle 6) Canadian sample was stratified by province/territory, type of school board (public *vs.* separate), urban–rural geographic status, school population size, and language of instruction (French vs. English). Children from private schools, home school, First Nations reserves, street youth, incarcerated youth, and youth not providing informed consent (explicit or implicit, as per school board customs) were excluded. Response rates were 11/13 (84.6%) at the province/territorial level, 436/765 (57.0%) at the level of schools, and 26,078/33,868 (77.0%) at the student level. Schools that refused participation were replaced by a neighboring school in the same board with similar demographic characteristics. The study was approved by the Queen’s University General Research Ethics Board.

### Measures

#### Material wealth

Self-perceived affluence or family wealth was measured in terms of a standard indicator of *relative material wealth* or affluence: *“how well off do you think your family is?”* (5 response options: “*very well off*” through “*not at all well off*”) [1]. For our positive and negative deviance analyses, we identified two groups for focused analyses based upon this measure: (1) students reporting the highest level of material wealth (“*very well off”*), and students reporting the lowest levels of material wealth (“*not very”, or “not at all”* well off).

#### General health status

The primary outcome was an item describing each child’s rating of their general health status: “*Would you say your health is?”: 1-“Excellent”; 2-“Good”; 3-“Fair”; 4-“Poor”*. Due to small cell sizes, the “fair” and “poor” categories were combined for some analyses. There is convincing evidence from large adolescent populations that such self-rated health measures represent relatively stable constructs over repeated observations during adolescence, and when using this measure, reported health deteriorates consistently with a lack of general well-being, disability, healthcare attendance and health-compromising behaviour, attesting to item validity [31].

#### Social support measures

A plethora of social support measures exist in the literature. Each largely assess different types (emotional, tangible, financial) and sources (parents, other caregivers, peers, teachers, other community members) of support [32]. We aligned more with the Forbes, et al. [8] conceptualization of social support that refers to interactions with family members, friends, neighbours, and peers which could provide instrumental, informational, and emotional support. For this reason, we measured social support using eight different items from the HBSC questionnaire that represent different types and sources of support from family, school, neighbourhood and peer-group contexts: ease of communication with parents; trust and understanding parents; frequency of family meals; level of family food security; neighbourhood social capital; involvement in group activities; and teacher and peer support at school.

#### Home and family measures

##### Ease of communication with parents

Participants were asked about communication in the home with parents or step-parents, i.e., “*How easy is it for you to talk to the following persons (categories included mother, father, step-mother, step-father) about things that really bother you*? “ (5 response options: *“very easy”, “easy”, “difficult”, “very difficult”, “don’t have or see this person”*) [22]. Those who found it easy to talk with at least one parent or step-parent were categorized separately from other children.

##### Parents trust and understanding

Each participant responded to four items about their home, with five response options ranging from *1-strongly agree* to *5-strongly disagree*: *“my parents understand me”; “I have a happy home life”; “my parents trust me”; “what my parents think of me is important”.* These items were combined into a factor analytically derived scale with very good psychometric properties (factor loadings 0.69 to 0.85; Cronbach’s alpha: 0.80) [26].

##### Family dinners

Each participant was asked *“On average, how many times per week does your family sit down at the table together for dinner/supper?”* (response options: *zero* through *7 times)* [22].

##### Food insecurity

Students’ perceptions of hunger due to food insecurity at home were measured: “*Some young people go to school or to bed hungry because there is not enough food at home. How often does this happen to you?*” with response options of *never, sometimes, often,* or *always*. This item has been subject to validation efforts internationally, and its relevance to the concepts of food insecurity and food poverty has been established [33].

#### Neighbourhood measures

##### Social capital

Neighbourhood social capital was assessed using a previously validated scale [22] that is based upon five items (*“people say hello and often talk to each other on the street”; “it is safe for younger children to play outside during the day”; “you can trust people around here”; “there are good places to spend your free time (i.e., recreation centres, parks, shopping centres*;” *“I could ask for help or a favour from neighbours”*)*,* with five response options ranging from *1-“strongly agree”* to *5-“strongly disagree”.* The combined scale based on these items had strong psychometric properties (Factor loadings: 0.59 to 0.75; Cronbach’s alpha: 0.75).

##### Involvement in group activities

Participants were asked if they were involved in the following groups or activities: *“sport club or team”; “voluntary service”; “church or religious group”; “youth club”; “cultural association (music, science, other)”.* Binary yes/no responses were combined into a simple index that ranged from 0 to 5 group activities.

#### School measures

##### School support

Existing scales describing the extent of *teacher support at school* and then *peer support by students at school* were used to describe supports and components of the overall social climate of school settings. These sub-scales are internally consistent, with factor loadings ranging from 0.62-0.77 for the teacher support (8 items, Cronbach’s alpha 0.87) and 0.71-0.83 for the peer support (5 items, Cronbach’s alpha 0.82) [26].

#### Demographic factors

Young people identified their *school grade* (≤*6* to ≥*10)* and gender (boy, girl)*.* Communities in which each participating school was located were classified according to Statistics Canada divisions of *urban–rural geography* [34]. These were *rural* (<1,000 population), *small urban centres* (1,000 to 19,999); *medium urban centres* (20,000 to 99,999); and *large urban centres* (≥100,000) [34]. A standard measure of *family structure* was used to describe the families of young people: *mother and father; mother and step-father; father and step-mother; mother only; father only; foster home; other family structure* [26]. Finally, young people reported whether they lived in one or two principle residences [35].

### Statistical analysis

Data analyses were conducted in IBM-SPSS Version 22 and SAS 9.3. In all analyses, standardized weights were applied to account for variations in sampling between provinces and territories. Descriptive analyses were used to characterize the study population demographically, first overall, then amongst young people reporting low and then high levels of affluence.

Next, we examined self-reported general health status (*excellent, good, fair, poor*) within the groups of young people with the lowest (n = 2,310) then highest (n = 5,501) reported levels of material wealth. This process was done overall within the two sub-samples, and then within groups defined by levels of each of the items and scales describing home (4 variables), neighbourhood (2 variables), and school supports (2 variables).

Logistic regression analyses were then conducted to model levels of health status as the dependent variable with each of the eight contextual support variables as independent variables. An affluence-social support interaction term was also included in the model. This analysis focused upon two groups that were positively then negatively deviant from norms: (1) young people from the lowest wealth backgrounds who reported *excellent* general health status (yes *vs.* no); (2) young people from the highest wealth backgrounds who reported low (*fair* or *poor*) general health status. Models accounted for the clustered nature of the sampling scheme with students nested within schools.

We next developed a simple cumulative index to describe the number of social supports (0 through 8) measured for each young person. Items contributing to this index were: (1) easy to communicate with at least one parent or step-parent; (2) high level (top quartile) of family trust and understanding; (3) always have family dinners; (4) never go to school or bed hungry; (5) high level (top quartile) of neighborhood social capital; (6) engagement in at least one group activity; (7) high level (top quartile) of teacher support at school; (8) high level (top quartile) of student support at school. In a series of four multiple logistic regression analyses, we then modeled the potential cumulative effects of this index on reports of poor and then excellent health in the two socio-economic groups of interest. Backwards elimination methods were used to identify a standard series of demographic confounders that were included in each of the four models. Effects are presented as adjusted odds ratios and associated 95% confidence limits. These sub-group analyses were powered to detect an increase in relative odds of 1.3 to 1.4 or higher when comparing health outcomes between quartiles, alpha = 0.05 (2 sided), dependent upon the subgroup and outcome of interest.

## Results

Overall, 26,078 young people were included in this study: 2,339 with low levels of material wealth and 5,738 with the highest levels of material wealth. The overall sample and these two sub-samples are profiled demographically in Table 1. There was a nearly even split of students by grade level and gender, with geographic distributions reflecting the weighting to ensure representativeness nationally. Substantial proportions of young people reported having a non-nuclear family structure and a second home, and these proportions were higher among those from less affluent circumstances.

**Table 1:** **Description of study population reporting low and then high family material wealth in the 2010 HBSC study for Canada**

**Variable**	**All respondents (n = 26,078)**		**Low material wealth (n = 2,339)**		**High material wealth (n = 5,738)**	
	**No.**	**(%)**	**No.**	**(%)**	**No.**	**(%)**
Grade						
≤6	5,165	(19.8)	529	(22.6)	1,322	(23.0)
7	5,205	(20.0)	450	(19.2)	1,361	(23.7)
8	5,266	(20.2)	439	(18.8)	1,159	(20.2)
9	5,395	(20.7)	488	(20.9)	1,000	(17.4)
≥10	5,047	(19.4)	433	(18.5)	897	(15.6)
*Not reported*	*0*		*0*		*0*	
Gender						
Boys	12,815	(49.1)	1,057	(45.2)	2,971	(51.8)
Girls	13,254	(50.8)	1,281	(54.8)	5,765	(48.2)
*Not reported*	*9*		*1*		*2*	
Urban–rural geographic status						
Rural	983	(3.8)	106	(4.6)	190	(3.3)
Small urban	10,767	(41.3)	960	(41.6)	2,268	(39.4)
Medium urban	5,739	(22.0)	490	(21.2)	1,290	(22.5)
Large urban	8,589	(32.9)	754	(32.6)	1,990	(34.7)
*Not reported*	*0*		*0*		*0*	
Family structure						
Both parents	16,965	(67.1)	1,186	(51.3)	4,407	(78.2)
Mother and stepfather	1,970	(7.8)	255	(11.0)	188	(5.1)
Father and stepmother	507	(2.0)	48	(2.1)	79	(1.4)
Mother only	3,824	(15.1)	535	(23.2)	47	(8.4)
Father only	815	(3.2)	126	(5.4)	20	(2.3)
Foster home	283	(1.1)	41	(1.8)	68	(1.2)
Other	908	(3.6)	120	(5.2)	193	(3.4)
*Not reported*	*805*		*28*		*101*	
Reports more than one home						
No	19,475	(78.6)	1,586	(70.1)	4,785	(86.0)
Yes	5,296	(21.4)	676	(29.9)	779	(14.0)
*Not reported*	*1,306*		*78*		*174*	

Table 2 describes the self-reported general health status of young people from homes with the lowest reported levels of material wealth or affluence. While the findings for individual items and scales are notable for their strength and statistical significance, what is most striking is the consistency of the observed findings. As the level of support available to students improved, reports of poor health status declined and reports of excellent health status consistently increased. This result occurred irrespective of the context for such supports, be that home, neighbourhood or school settings. Clearly, even in socio-economically disadvantaged circumstances, the presence of social supports was associated with improved perceptions of health. These exploratory analyses were extended in Table 3 to focus on young people from the highest levels of material wealth. The primary outcome here was a report of “fair” or “poor” health. As the degree of support available from home, neighborhood and school contexts diminished, reports of poor health increased. Clearly, even in socio-economically advantaged circumstances, absence of social supports in homes, neighborhoods, and schools was associated with higher perceived levels of poor health.

**Table 2:** **General health status of young Canadians by family, neighborhood, school and peer group characteristics (respondents limited to the 2,310 with low material wealth)**

**Factor**	**No.**	***Percent (row) reporting health status that is:***					***Excellent*** **health**
		**Poor**	**Fair**	**Good**	**Excellent**	**p-value**	**OR (95% CI)**
**All respondents – No.**	2,310	6.3	19.7	51.2	21.6		
**Parents/step-parents in home that are “easy” or “very easy” to talk to:**							
None	695	11.5	23.3	49.6	15.5	<0.0001	1.00
At least one	1,583	4.1	18.5	53.3	24.1		1.73 (1.37 to 2.19)
**Parents trust and understanding scale:**							
Q1 (lowest)	1,064	10.1	25.2	50.5	14.3	<0.0001	1.00
Q2	432	3.2	17.4	58.1	21.3		1.62 (1.22 to 2.16)
Q3	296	5.4	20.3	52.0	22.3		1.73 (1.25 to 2.39)
Q4 (highest)	371	0.5	7.3	48.0	44.2		4.77 (3.64 to 6.22)
**Family dinners eaten together per week:**							
None	373	12.1	29.0	41.0	18.0	<0.0001	1.00
1 to 3 times	595	5.7	21.2	59.7	13.4		0.70 (0.49 to 1.01)
4 to 6 times	629	4.5	19.6	53.4	22.6		1.33 (0.96 to 1.83)
Every day	584	5.5	13.0	49.1	32.4		2.18 (1.56 to 2.99)
**Go to bed or school hungry because of not enough food in the home:**							
Always	64	20.3	28.1	35.9	15.6	<0.0001	1.00
Often	169	7.1	27.2	54.4	11.2		0.73 (0.32 to 1.67)
Sometimes	745	7.0	25.5	50.7	16.8		1.11 (0.56 to 2.33)
Never	1,313	5.4	15.3	52.8	26.5		2.05 (1.02 to 4.11)
**Neighborhood social capital scale:**							
Q1 (lowest)	811	8.9	27.1	48.0	16.0		1.00
Q2	608	5.9	16.8	56.1	21.2		1.41 (1.08 to 1.85)
Q3	382	3.1	18.1	51.8	27.0		1.94 (1.45 to 2.60)
Q4 (highest)	355	3.7	8.5	54.6	33.2	<0.0001	2.61 (1.95 to 3.49)
**Participate in group activities:**							
None	830	10.1	28.1	47.7	14.1	<0.0001	1.00
1	638	4.9	17.7	50.3	17.1		2.26 (1.74 to 2.94)
2	354	1.1	12.4	65.5	20.9		1.60 (1.16 to 2.21)
3 or more	330	4.5	9.4	51.8	34.2		3.16 (2.34 to 4.26)
**Support from teachers at school scale:**							
Q1 (lowest)	796	8.5	24.2	47.9	19.3	<0.0001	1.00
Q2	586	6.5	24.1	51.7	17.7		0.90 (0.68 to 1.18)
Q3	415	5.5	13.3	56.4	24.8		1.37 (1.03 to 1.82)
Q4 (highest)	382	3.1	11.5	57.3	28.0		1.62 (1.22 to 2.15)
**Support from other students at school scale:**							
Q1 (lowest)	942	9.1	26.3	47.6	17.0	<0.0001	1.00
Q2	409	5.6	22.5	53.8	18.1		1.08 (0.79 to 1.46)
Q3	474	4.6	13.3	59.5	22.2		1.38 (1.05 to 1.82)
Q4 (highest)	419	2.9	11.7	49.4	36.0		2.73 (2.10 to 3.55)

*Note: Tests for linear trend were statistically significant p < 0.0001.*

**Table 3:** **General health status of young Canadians by family, neighborhood, school and peer group characteristics (respondents limited to those with high material wealth)**

**Factor**	**No.**	***Percent (row) reporting health status that is:***					***Poor*** **health**
		**Poor**	**Fair**	**Good**	**Excellent**	**p-value**	**OR (95% CI)**
**All respondents**	5,551	1.3	7.9	43.9	46.9		
**Parents/step-parents in home that are “easy” or “very easy” to talk to:**							
At least one	4,999	0.8	7.0	43.5	48.7	<0.0001	1.00
None	552	5.3	15.8	47.6	31.3		3.13 (2.49 to 3.94)
**Parents trust and understanding scale:**							
Q4 (highest)	2,232	0.4	5.3	37.6	56.7	<0.0001	1.00
Q3	1,335	0.7	6.3	47.0	46.0		1.24 (0.94 to 1.63)
Q2	1,022	1.5	9.8	51.9	36.9		2.07 (1.59 to 2.70)
Q1 (lowest)	717	4.9	16.2	45.7	33.2		4.40 (3.42 to 5.67)
**Family dinners eaten together per week:**							
Every day	2,374	0.6	6.5	41.4	51.5	<0.0001	1.00
4 to 6 times	1,925	1.1	6.7	45.7	46.4		1.12 (0.89 to 1.40)
1 to 3 times	873	1.6	12.1	47.3	38.6		2.07 (1.62 to 2.66)
Never	249	7.2	12.4	41.8	38.6		3.23 (2.28 to 4.58)
**Go to bed or school hungry because of not enough food in the home:**							
Never	4,632	1.1	7.4	43.0	48.6	<0.0001	1.00
Sometimes	853	1.2	9.5	48.1	41.3		1.28 (1.01 to 1.63)
Often	89	4.5	12.4	43.8	39.3		2.19 (1.24 to 3.86)
Always	60	16.7	6.7	41.7	35.9		3.23 (1.75 to 5.96)
**Neighbourhood social capital scale:**							
Q4 (highest)	1,717	1.0	5.2	36.3	57.5	<0.0001	1.00
Q3	1,158	0.9	5.6	45.9	47.6		1.07 (0.79 to 1.45)
Q2	1,412	1.0	9.4	49.4	41.2		1.77 (1.36 to 2.30)
Q1 (lowest)	1,028	3.0	11.9	48.0	37.2		2.67 (2.05 to 3.46)
**Participate in group activities:**							
3 or more	1,457	2.6	13.6	50.2	33.6	<0.0001	1.00
2	2,028	1.0	5.3	39.8	53.9		0.46 (0.31 to 0.66)
1	1,050	0.8	3.7	43.9	51.6		0.66 (0.50 to 0.89)
None	879	0.5	8.6	41.9	49.0		1.92 (1.47 to 2.51)
**Support from teachers at school scale:**							
Q4 (highest)	1,691	0.9	4.7	37.9	56.4	<0.0001	1.00
Q3	1,243	0.1	7.8	46.1	46.0		1.41 (1.05 to 1.89)
Q2	1,258	1.2	9.1	47.3	42.2		1.89 (1.44 to 2.49)
Q1 (lowest)	1,110	3.2	10.7	46.1	39.9		2.70 (2.06 to 3.51)
**Support from other students at school scale:**							
Q4 (highest)	1,685	0.9	4.7	37.4	56.9	<0.0001	1.00
Q3	1,457	0.8	6.0	47.4	45.9		1.19 (0.89 to 1.59)
Q2	1,001	0.2	8.3	46.4	45.2		1.53 (1.13 to 2.07)
Q1 (lowest)	1,375	3.1	12.6	47.0	37.3		3.09 (2.40 to 3.97)

*Note: Tests for linear trend were statistically significant p < 0.0001.*

**Figure 1:**
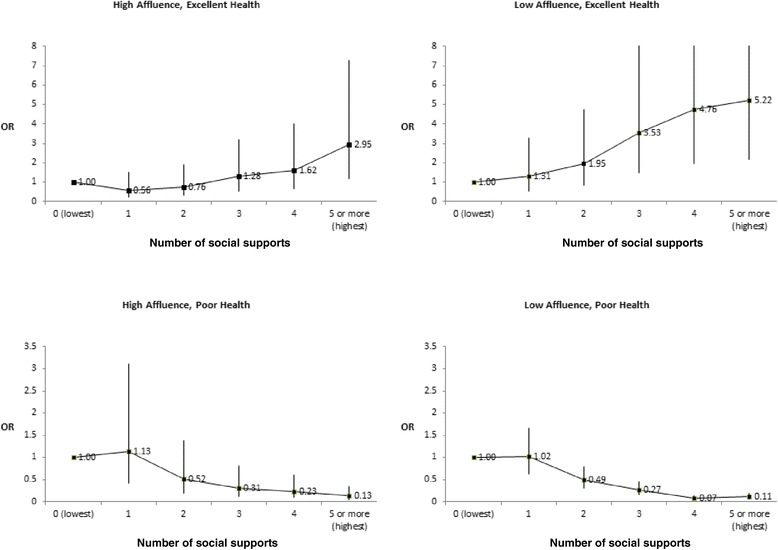
**Adjusted relative odds (95% CI) of self-reported health (excellent or poor) by number of social supports (0 to 5 or more).** Note: Analyses were conducted by level of material wealth (high affluence, n = 5,541; low affluence, n = 2,310). Findings were stratified by level of affluence as it was found to be an effect modifier in the relationship between social supports and self-perceived health. All analyses were adjusted for gender, grade level, and family structure. Tests for linear trend were statistically significant (p < 0.0001).

Figure 1 graphically shows the remarkable strength, consistency and dose-dependent nature of these associations when based upon our cumulative social support index. These analyses were adjusted for grade level, gender, and family structure. In the test for interaction between level of affluence and social support, we found that relations between number of social supports and excellent health were significantly stronger (p < 0.0001) among low affluent vs. high affluent children, although supports mattered for both groups of children. In addition, relations between number of social supports and poor health were significantly (p < 0.0001) more protective for children from low vs. high affluence backgrounds, although again supports mattered for both groups of children. Among the most affluent young people, an almost 3-fold increase in the relative odds of “excellent” health was reported by young people with the highest numbers of social supports relative to those with no reported social supports. This gradient was even more striking in the low affluence group, where the observed increases in odds ratios were 5-fold and followed a similar social gradient. While both overall patterns were similar, it appears the association stronger among low affluence groups. We do recognize that because the wealthier were more likely to have excellent health in all categories there is less scope for increased odds ratios as social supports increase. In terms of absolute difference the benefit may not be so obviously greater among the low affluent group when this is considered. The bottom two graphs depict the models describing an outcome of poor general health status. As the levels of social support increased, the relative odds of reporting poor health decreased, again in a dose-dependent manner. A test for interaction between level of affluence and social support was statistically significant (p < 0.0001).

## Discussion

This study of a large national sample of Canadian adolescents reaffirms a significant amount of past research (e.g., [36]; [11] that demonstrates that social supports are important to the health of young people. First, we examined eight available types and sources of social support, two kinds in each family, neighbourhood, school and peer group contexts. We derived a simple additive scale describing cumulative supports for adolescents in low and high affluence situations. Next, we focused specifically on young people who deviated from an expected health-wealth relationship. We were interested in the social supports of young people who are from low affluence situations and yet are thriving in terms of self-reported health, as well as those from high affluence situations who have low perceived health. Our analyses included adjustment for sex, grade level, and different family structures and an interaction term for social support and affluence. Our findings indicate that social supports in all eight domains were associated with adolescent self-perceived health and there was an interactive effect between affluence and social support. A direct, consistent and dose-dependent relationship exists, for both high and low levels of affluence. We believe the most striking finding is the *cumulative* potential effects of social supports. Reported accumulation of numerous social supports was shown to be beneficial in both groups and this relationship was especially pronounced among those who were in the lowest wealth categories.

All eight aspects of social support that were included in the scale had a positive impact on self-reported health. When examined individually, relations between parental trust and understanding and participation in group activities were particularly strong. Peer support is an established protective factor for improved adolescent health ([36]; [11]. Such support is often beneficial on its own, but is also associated with involvement in group activities and therefore may indirectly contribute to health via the benefits of group activities as well as increased opportunities for adult mentorship [25].

Parental support at home, as measured by scales related to trust and communication, eating meals together and providing food was also beneficial to health. The manner in which parents guide, show care and warmth or provide structure for young people has an important influence on their levels of attachment, security and perceived well-being [37]. Further, variations in parenting styles (permissive, effective, authoritarian and uninvolved) have been shown to help to explain deviation from norms in expected health outcomes [38,39]. Adolescents tend to be healthier, happier and better adjusted in families that maintain consistent expectations and open lines of communication, where the parents are neither punitive nor aloof but rather affectionate and involved in their children’s lives [40,41]. The influence of these fundamental determinants of health were strongly reflected in our findings. Increased trust and communication with adults in the home was associated with improved health in young people in both groups, but particularly in those from low affluence situations. Conversely, when these family qualities were absent, this was reflected in lower self-perceived health.

In addition to a supportive peer group and family, other supportive adults can also help young people to process early emotional experiences and build inner personal qualities and intelligence, and these in turn help them build resilience and cope with adversity [37]. Our findings suggest that young people who have other caring adults in their lives report more positive health largely irrespective of the roles these adults might take in terms of being teachers, neighbours, coaches or others [42]. Indeed, we found that neighbourhood social capital, the support of teachers, and group involvement (which may be connected to coaches and other respected leaders) all contributed to better self-reported health states in young people.

In this study we have measured self-perceived health and self-perceived social support. Barker [11] argues that “objective measures of social support are generally less important (and perhaps less valid) than the subjective meaning that an adolescent attributes to these social supports” (p. 3). This suggests that self-report measures may in actuality be a good way of capturing the relationship between social support and overall health. We do have to acknowledge that this was the approach we used, however, when we are interpreting findings.

It is clear that social supports matter for young people. Our study affirms in a convincing matter that cumulative social supports may influence child health in a positive manner at both ends of the affluence spectrum. While the findings were most pronounced in the low affluence group; decreases in supports within even the high affluence group were clearly associated with detrimental health consequences. The cumulative effects of these supports, or lack thereof, were profound. The strength and consistency of the dose–response relationships observed were notable, be they the positive cumulative influences of social supports on health, or the negative cumulative effect of the absence of such supports. With respect to our original idea of exploring factors underlying unexpected health states as a means to inform health interventions, the data clearly indicate that social supports matter. We did not investigate the comparative influence of different types of social supports (family versus peer or teacher, for example) and this might be a useful follow-up study. However, we believe this study helps us affirm that beyond socioeconomic influences, for children at different ends of the affluence continuum social supports are a salient part of understanding positive health etiology.

## Conclusions

We are reminded of the well-known proverb: it takes a village to raise a child. The origin of this exact proverb is not clear or substantiated; however, many cultures, especially in continental Africa, profess a similar idea, i.e., regardless of a child’s biological parentage, its upbringing is not the responsibility of one parent or home but the responsibility of the whole community. While a strong parent and home life is optimal for children, the negative effects appear to be mediated by other supports if that support is missing. Clearly, for children to have good health, they need to have people (and other supports) available on whom they can rely, and who can let them know tangibly that they are cared about, valued, and loved. A caring teacher, regular family meals or a good friend; access to wealth may offer health to some, but the accumulation of social support is an important key to health as well.

Strengths of our study warrant comment. They include the size and diversity of the study population, as well as its national scope. The analysis itself and its emphasis on cumulative effects was novel and addressed several practical questions surrounding social supports as mechanisms which explain common socio-economic gradients in health. As well as providing evidence in support of a number of social theories, the analysis also provided direction for preventive interventions. Limitations include the cross-sectional HBSC design, and any causal inferences that were made require confirmation in longitudinal analyses. Further, the HBSC sampling strategy excluded some high-risk adolescents in non-classroom settings (e.g., incarcerated youth, youth living on First Nations Reserves, home schooled and private school students), which may impact upon the external validity of our findings. In addition, we recognize the potential for response bias in the HBSC. Although HBSC in Canada has shown to be representative of Canadian school-aged children in terms of basic demographics (e.g., those described in Table 1), it is possible that the prevalence levels of certain behaviours are over- or under-represented in the realized sample due to non-response during recruitment. This remains a limitation of any self-report survey that relies upon volunteer school systems and students. The main point of our analyses were to study relations between exposures (social supports) and outcomes (e.g. self-perceived health status). While, hypothetically, there may be slight differences in the heterogeneity of our sample in terms of these exposures compared with Canada as a whole, it is very unlikely that true selection bias occurred where relationships between social supports and health are distorted due to sampling and recruitment.

In conclusion, this novel study of young Canadians demonstrated that cumulative levels of social support are important to health, irrespective of socio-economic background. While family and peer group supports are especially salient, other supports obtained from neighbourhood and school social contexts can also be critical and can compensate in part for dysfunctional social environments at home. We believe that this shared sense of responsibility for child health is significant and that we can potentially all play a role in its promotion. Further, we reaffirm the idea that it just may take a village to raise a healthy child.

## Abbreviations

HBSC: Health Behaviour in School-aged Children
WHO: World Health Organization
